# The Notch signaling pathway controls CD8^+^ T cell differentiation independently of the classical effector HES1

**DOI:** 10.1371/journal.pone.0215012

**Published:** 2019-04-05

**Authors:** Dave Maurice De Sousa, Frédéric Duval, Jean-François Daudelin, Salix Boulet, Nathalie Labrecque

**Affiliations:** 1 Maisonneuve-Rosemont Hospital Research Center, Montréal, Québec, Canada; 2 Département de microbiologie, infectiologie et immunologie, Université de Montréal, Montréal, Québec, Canada; 3 Département de médecine, Université de Montréal, Montréal, Québec, Canada; Univerzitet u Beogradu, SERBIA

## Abstract

During CD8^+^ T cell response, Notch signaling controls short-lived-effector-cell (SLEC) generation, but the exact mechanisms by which it does so remains unclear. The Notch signaling pathway can act as a key regulator of Akt signaling via direct transcriptional induction of *Hes1*, which will then repress the transcription of *Pten*, an inhibitor of Akt signaling. As both Notch and Akt signaling can promote effector CD8^+^ T cell differentiation, we asked whether Notch signaling influences SLEC differentiation via the HES1-PTEN axis. Here, we demonstrate that HES1 deficiency in murine CD8^+^ T cells did not impact SLEC differentiation. Moreover, we show that *Pten* transcriptional repression in effector CD8^+^ T cells is not mediated by Notch signaling although Akt activation requires Notch signaling. Therefore, HES1 is not an effector of Notch signaling during CD8^+^ T cell response.

## Introduction

CD8^+^ T cells are essential for the successful elimination of several infectious agents and are endowed with the ability to control tumor growth. We, and others, have recently discovered that Notch signaling is central to the proper differentiation of CD8^+^ effector cells [1,2]. Notch deficiency severely impairs the generation of short-lived effector T cells (SLECs) during acute response to infection and vaccination [1,2]. Following ligand engagement, the intracellular domain of Notch (NICD) translocates to the nucleus where it associates with RBPJk to induce the transcription of common (e.g. *Hes1*) and tissue-specific target genes. The relative contribution of the common versus tissue-specific Notch target genes to effector CD8^+^ T cell differentiation is still unknown. However, the requirement for Notch-mediated HES1 induction during T-lineage commitment and thymocyte β-selection raises the possibility that Notch signaling controls effector CD8^+^ T cell differentiation via *Hes1* transcriptional induction [3,4].

One key event controlling effector and SLEC differentiation is the activation of the Akt-mTOR pathway, which mediates the metabolic switch from catabolism to anabolism necessary for differentiation [5–10]. Furthermore, sustained and strong Akt activation in CD8^+^ T cells enhances effector function and promotes SLEC differentiation [6,8]. Interestingly, Notch signaling controls the activation of Akt and mTOR in thymocytes and T lymphoblastic leukemias (T-ALL) [4,11,12]. The activation of Akt can be mediated by transcriptional induction of the common Notch target gene *Hes1* [4]. One mechanism that has been described proceeds via HES1 mediated transcriptional repression of *Pten*, an inhibitor of Akt activation [4]. The need for proper activation of the Akt-mTOR and Notch signaling pathways for SLEC differentiation raises the possibility that Notch signaling promotes SLEC differentiation via the induction of the common effector HES1, which then represses *Pten* transcription allowing for proper activation of the Akt signaling pathway. Using mice lacking expression of HES1 in mature CD8^+^ T cells, we show that HES1 induction by Notch is not necessary for effector CD8^+^ T cell differentiation. Furthermore, we show that unlike in thymocytes and T-ALL, the Notch signaling pathway does not repress *Pten* transcription. However, even if *Pten* transcription is repressed efficiently in absence of Notch and HES1, the Akt-mTOR pathway is not properly activated during CD8^+^ T cell response in the absence of Notch signaling while HES1 deficiency has no effect.

## Materials and methods

### Mice

*Notch1*^fl/fl^/*Notch2*^fl/fl^ OT-1 *Rag1*^-/-^ and E8I-cre^+/-^ *Notch1*^fl/fl^*Notch2*^fl/fl^ OT-1 *Rag1*^-/-^ mice were previously described [1]. *Hes1*^fl/fl^ mice were a kind gift from Dr. Ryoichiro Kageyama [13]. *Hes1*^fl/fl^ were backcrossed for at least 10 generations to C57BL/6 mice and were bred with E8I-cre mice [14] to obtain E8I-Cre^+/-^*Hes1*^fl/fl^ (Δ/Δ; lacking HES1 expression only in mature CD8^+^ T cells) and *Hes1*^fl/fl^ (Hes1-sufficient, fl/fl). E8I-cre^+/-^*Hes1*^fl/fl^ mice were also bred to OT-I *Rag1*^-/-^ mice [15]. B6.SJL mice were bred in house. All mice were bred and housed in a pathogen-free environment under conventional conditions at room temperature of 22–25°C with acidified water *ad libitum*. Mice were feed Teklad global 18% protein diet (Envigo), given environmental enrichment (Nestlets, tunnels and igloos) and treated in accordance to the Canadian Council on Animal Care guidelines. Following infection, mice were monitored daily for weight loss, dehydration and lethargy. Our animal protocol (number: 2017AV010) was approved by the Hospital Maisonneuve-Rosemont Council on Animal Care.

### Analysis of OVA-specific CD8^+^ T cell response

For analysis of T cell response, mice were injected i.v. with a sublethal dose of 2 X 10^3^ CFU *Listeria monocytogenes* expressing OVA (Lm-OVA) as previously described [16]. B6.SJL bone marrow derived dendritic cells were matured with LPS (1 μg/ml), and loaded with the ovalbumin peptide (SIINFEKL; OVA_257–264_ 2 μg/ml; Midwest biotech) (DC-OVA) as previously described [17]. 1.25 x 10^6^ DC-OVA were injected i.v for immunization. *Ex vivo* primary endogenous CD8^+^ T cell response analysis was performed on spleen at day 7 post-infection or vaccination. In experiments using adoptive transfer of OT-I T cells of different genotypes, 10^6^ cells were transferred into B6.SJL recipient mice followed by Lm-OVA infection. OT-I T cell response was analyzed in the spleen at day 3 post-infection.

### Abs, flow cytometry and cell sorting

Anti-CD8 (53–6.7), anti-CD44 (IM7), anti-KLRG1 (2F1), anti-CD127 (A7R34) and anti-CD45.2 (104) Abs were from Biolegend; anti-IFN-γ (XMG1.2) Ab was from Life Technologies; anti-TNF-α, anti-p-S6 (CUPK43K) and anti-p-AKT_S473_ (SDRNR) Abs were from eBioscience; anti-p-Akt_T308_ (13038) was from Cell Signaling Technology. Cell surface, intracellular and tetramer stainings were performed as previously described [17–19]. For analysis of p-Akt_S473,_ and p-S6, splenocytes were rested in RPMI 1% FCS and then stimulated for 1h with the OVA peptide followed by fixation, permeabilization and staining using the BD cytofix/cytoperm reagent. For analysis of p-Akt_T308_, splenocytes were rested in RPMI 1% FCS and the stimulated for 1h with the OVA peptide (2 μg/mL) followed by fixation, permeabilization and staining using the eBioscience Foxp3 staining kit. A second step staining was performed with polyclonal goat anti-rabbit IgG (H+L) highly cross-adsorbed secondary antibody Alexa Fluor Plus 647 from ThermoFischer (#A32733) to reveal p-Akt_T308_ staining. In some experiments, the level of p-Akt and p-S6 was measured directly *ex vivo*. Naïve CD8^+^ T cells (CD8^+^CD44^low^), day 7 OVA-specific CD8^+^ effector T cells (CD8^+^CD44^high^Tet-OVA^+^) and day 3 OT-I effector CD8^+^ T cells (CD8^+^CD45.2^+^CD44^high^) were sorted with a BD FACSARIA III.

### RT-qPCR

Quantification of *Hes1* and *Pten* mRNAs from sorted OT-I CD8^+^ T cells was performed as previously described [19,20]. Sequences of primers used were as follows: *Hes1*, 5’- ATAGCTCCCGGCATTCCAAG -3’ and 5’-GCGCGGTATTTCCCCAACA-3’; *Pten*, 5’-GAGTATCTTGTACTCACCCTAAC-3’ and 5’-GGATTTGATGGCTCCTCTAC-3’.

### Statistical analysis

Statistical analyses for differences between the fl/fl and Δ/Δ groups were done using Student’s T test. Welch’s correction was applied for unequal variances when required. ANOVA was used when comparing more than two experimental groups. Tukey’s correction was applied for unequal variances when required. Data are presented as mean +/- standard error of the mean (SEM). Only significant statistical differences are indicated on the figures.

## Results and discussion

### Notch-dependent induction of *Hes1* transcription in antigen-specific CD8^+^ T cells

**Fig 1:**
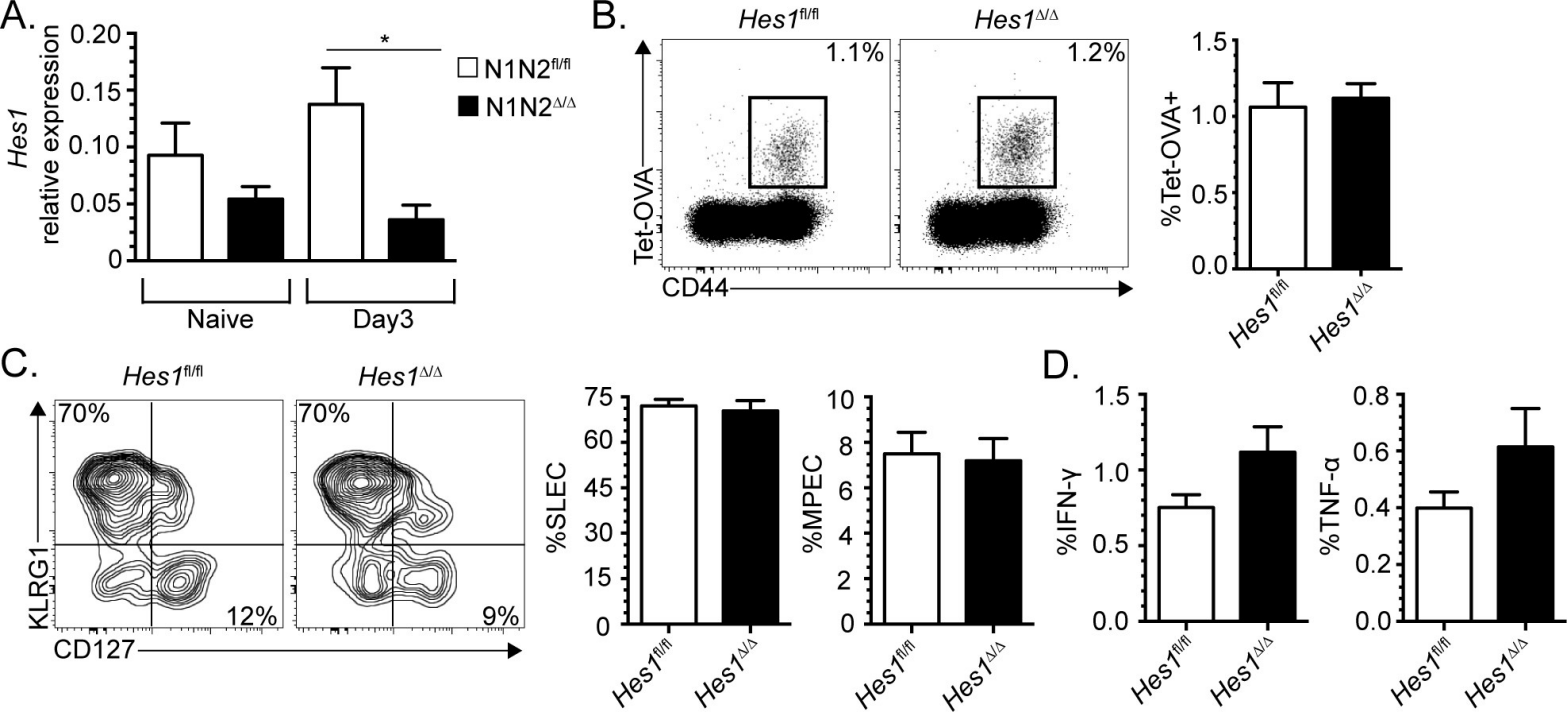
HES1 is dispensable for effector CD8^+^ T cell differentiation following infection with *Listeria monocytogenes*. (A) 10^6^ WT or Notch1/2-deficient OT-I CD8^+^ T cells were adoptively transferred in congenic B6.SJL mice followed by infection with Lm-OVA. At day 3 post-infection, OT-I CD44^high^ T cells were sorted to analyze *Hes1* transcription by RT-qPCR. (B-D) HES1-sufficient (fl/fl) and -deficient (Δ/Δ) mice were infected with Lm-OVA and CD8^+^ T cell response was analyzed at day 7 in the spleen. (B) OVA-specific CD8^+^ T cell response was identified using tetramer (K^b^-OVA; Tet-OVA) and anti-CD44 antibody stainings. The percentage of OVA-specific CD8^+^ T cells is indicated on each profile (gated on CD8^+^ T cells). The compilation of the percentage of OVA-specific CD8^+^ T cells is shown next to the FACS profiles. (C) FACS profiles and compilation of SLECs (KLRG1^+^CD127^-^) and MPECs (KLRG1^-^CD127^+^) among OVA-specific CD8^+^ T cells (Tet-OVA^+^CD44^+^). (D) Cytokine production by OVA-specific CD8^+^ effectors after a short (5h) *in vitro* restimulation with the OVA peptide. Data are representative of two (A) or five (B-D) independent experiments with 2–3 mice per group. Statistical significance was determined using ANOVA (A) and Student’s t test (B-D). *p<0.05.

Following ligand engagement, Notch receptors are cleaved to generate the NICD that will then migrate to the nucleus to induce gene transcription. Among the induced genes are classical effector of the Notch signaling pathway such as *Hes1* and *Dtx1*. As *Hes1* transcriptional induction by the NICD was shown to control critical aspects of thymic T cell differentiation [3,4], we evaluated whether *Hes1* transcription is induced following *in vivo* Notch signaling in Ag-specific CD8^+^ T cells. We adoptively transferred 10^6^ wild-type or Notch1/2-deficient OT-I TCR transgenic CD8^+^ T cells [1], specific for the ovalbumin (OVA) peptide in the context of K^b^, into congenic B6.SJL recipients followed by infection with a recombinant strain of *Listeria monocytogenes* encoding OVA (Lm-OVA). Three days later, WT and Notch1/2 deficient OT-I CD8^+^ T cells were sorted to measure *Hes1* transcription. As shown in Fig 1A and S1A Fig, *Hes1* transcription was induced in WT but not in Notch1/2-deficient OT-I T cells. This induction of *Hes1* transcription is less than what has been described by the Immunological Genome Project Consortium (immgen.org) [21] in thymocytes receiving Notch signal (8-fold higher in DN2/3 thymocytes compared to DP or SP thymocytes) but suggests that *Hes1* transcriptional induction may contribute to CD8^+^ T cell differentiation.

### CD8^+^ T cell response to *Listeria* infection is not affected by HES1-deficiency

Considering that the Notch signaling pathway controls SLEC differentiation [1,2], we asked whether this occurs via the Notch target gene *Hes1*. We crossed *Hes1*-floxed mice with E8I-cre mice to generate mice in which the *Hes1* gene is specifically deleted in mature peripheral CD8^+^ T cells (referred as *Hes1*^Δ/Δ^) [13,14]. Mice were then infected with Lm-OVA and the OVA-specific CD8^+^ T cell response was analyzed at day 7 post-infection. HES1-deficiency in CD8^+^ T cells did not affect T cell expansion (Fig 1B) and had no effect on the SLEC/MPEC differentiation choice (Fig 1C). The lack of effect on SLEC differentiation was not due to overgrowth of non-deleted cells (S1B Fig). Furthermore, the HES1-deficient effectors generated are functional as shown by their ability to produce IFN-γ and TNF-α (Fig 1D). Therefore, HES1 induction by Notch signaling is not involved in SLEC differentiation and acquisition of effector functions following *Listeria* infection.

### HES1-deficient CD8^+^ T cells respond normally to DC vaccination

**Fig 2:**
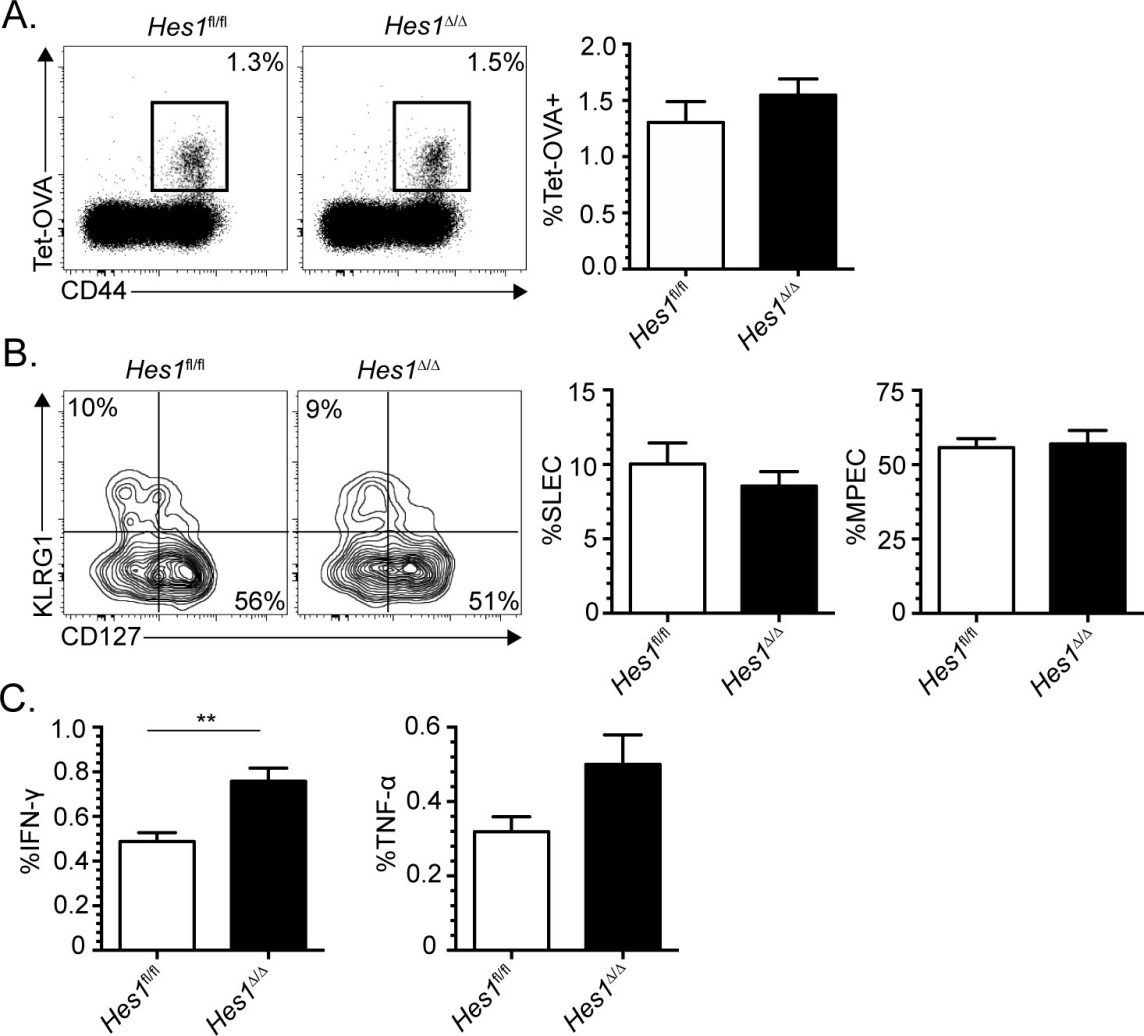
HES1 deficiency does not affect effector CD8^+^ T cell differentiation following dendritic cell vaccination. *Hes1*^fl/fl^ and *Hes1*^Δ/Δ^ mice were vaccinated with DC-OVA and CD8^+^ T cell response was analyzed at day 7 in the spleen. (A) OVA-specific CD8^+^ T cell response was identified using tetramer (K^b^-OVA; Tet-OVA) and anti-CD44 antibody stainings. The percentage of OVA-specific CD8^+^ T cells is indicated on each FACS profiles (gated on CD8^+^ T cells). The compilation of the percentage of OVA-specific CD8^+^ T cells is shown next to the FACS profiles. (B) FACS profiles and compilation of SLECs (KLRG1^+^CD127^-^) and MPECs (KLRG1^-^CD127^+^) among OVA-specific CD8^+^ T cells (Tet-OVA^+^CD44^+^). (C) Cytokine production by OVA-specific CD8^+^ effectors after a short (5h) *in vitro* restimulation with the OVA peptide. Data are representative of three independent experiments with 2–3 mice per group. Statistical significance was determined using Student’s t test.

We have previously demonstrated that Notch signaling plays a context dependent role. Following infection, which induces a high level of inflammation, Notch signaling was dispensable for the acquisition of effector functions while it was critical in the low inflammation setting of dendritic cell (DC) vaccination [1]. Furthermore, SLEC generation was more severely affected following DC vaccination than infection [1,2]. Therefore, we evaluated whether the induction of HES1 was important for the differentiation of CD8^+^ T cells following DC vaccination. Even in the context of low inflammation, *Hes1* transcriptional induction was not required for the differentiation of SLECs and acquisition of effector functions (Fig 2). The lack of requirement for HES1 during SLEC differentiation during *in vivo* CD8^+^ T cell activation even if *Hes1* is transcriptionally induced by Notch signaling is similar to what has been observed during the differentiation of marginal zone B cells [3]. Furthermore, it illustrates that Notch signaling relies on the induction of different direct target gene(s) depending on the differentiation context.

### The Notch signaling pathway does not influence *Pten* transcription in effector CD8^+^ T cells

**Fig 3:**
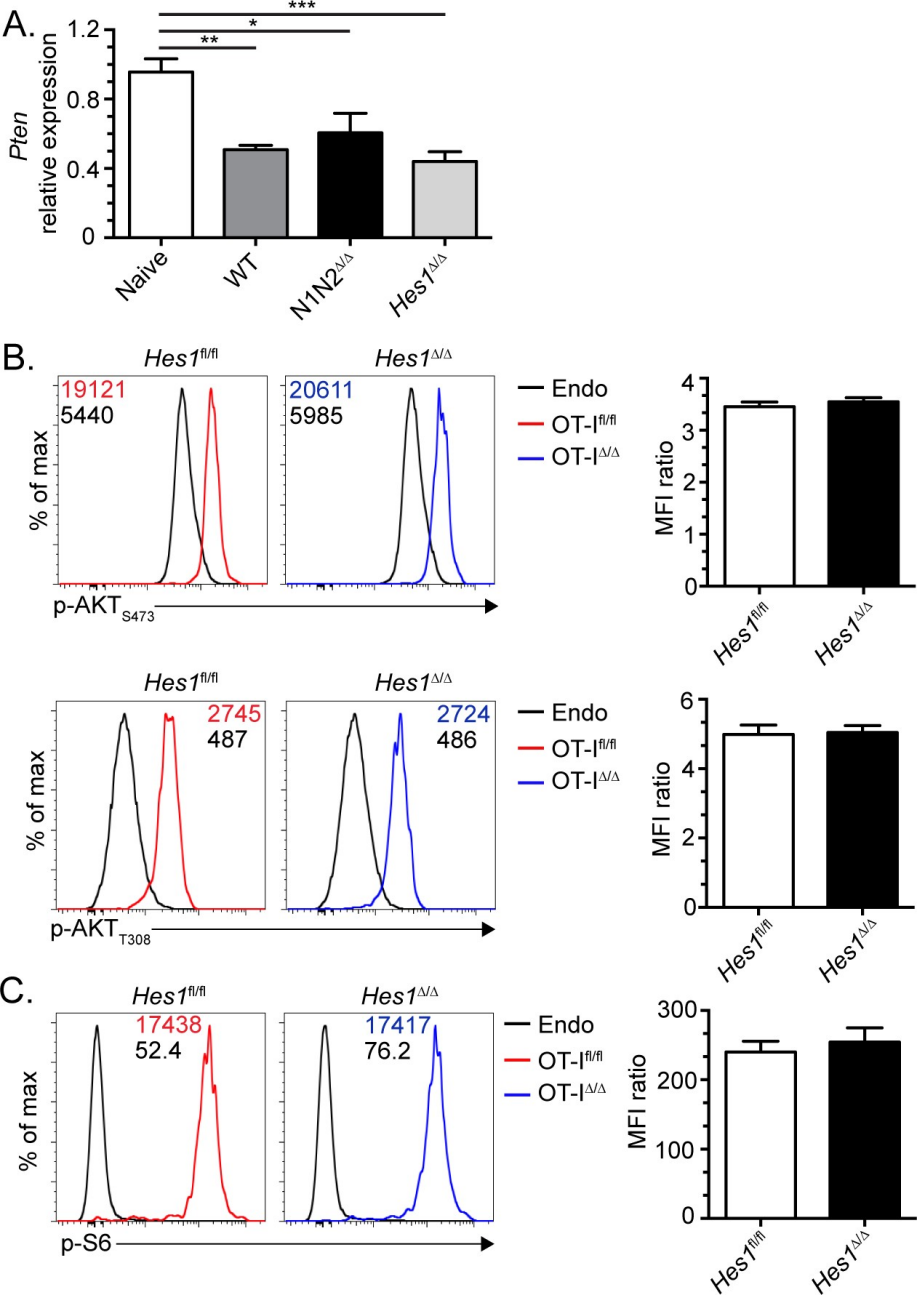
The Notch signaling pathway does not control *Pten* transcription in effector CD8^+^ T cells. (A) 10^6^ WT, Notch1/2-deficient or HES1-deficient OT-I CD8^+^ T cells were adoptively transferred in congenic B6.SJL mice followed by infection with Lm-OVA. At day 3 post-infection, OT-I CD44^high^ T cells (CD8^+^CD45.2^+^) were sorted to analyze *Pten* transcription by RT-qPCR. Naïve OT-I T cells were sorted before the adoptive transfer. (B-C) WT and HES1-deficient OT-I CD8^+^ T cells were adoptively transferred in B6.SJL mice followed by infection with Lm-OVA. At day 3 post-infection, cells were briefly (1h) restimulated with OVA peptide (2 μg/mL) before intracellular staining to detect phospho-Akt (B) and phospho-S6 (C) in OVA-specific OT-I effectors (CD8^+^CD45.2^+^). Endogenous CD8^+^ T cells from the same recipient mice (CD8^+^CD45.2^-^) were used as a staining control. The bar graphs show the ratio of the MFI of OVA-specific CD8^+^ T cells over the endogenous CD8^+^ T cells. Two independent experiments with 5 mice per group. Statistical significance was determined using ANOVA (A) and Student’s t test (B-C). *p<0.05, **p<0.01 and ***p<0.001.

The induction of *Hes1* transcription has been shown to be essential for the repression of *Pten* transcription in thymocytes to promote their survival, differentiation and proliferation at the β-selection checkpoint [4]. This repression of *Pten* transcription is necessary to permit the activation of the Akt signaling pathway [4]. As the activation of Akt is also an essential event for the differentiation of SLECs [5–9], it was surprising that HES1 expression was not necessary for SLEC differentiation. Therefore, we tested whether *Pten* transcriptional repression occurs during *in vivo* CD8^+^ T cell activation and if this was regulated by Notch signaling. We sorted WT, Notch1/2-deficient and *Hes1*^Δ/Δ^ OT-I naïve and day 3 effectors. *Pten* transcription was lower in day 3 effectors than in naïve OT-I T cells and was similarly reduced in absence of Notch signaling and HES1 (Fig 3A). Altogether, these results indicate that the repression of *Pten* transcription occurs independently of the Notch signaling pathway in mature peripheral CD8^+^ T cells. This is similar to what has been observed in absence of Notch signaling in T cells during graft versus host disease [22].

Although *Pten* transcription was repressed as efficiently in WT and Notch1/2-deficient effectors, it was reported by the group of Amsen that Notch-deficiency in Ag-specific CD8^+^ T cells led to reduced activation of the Akt signaling pathway [2]. We also observed reduced phosphorylation of Akt in Notch-deficient CD8^+^ T cells (S1C Fig). However, HES1 induction was not required for proper phosphoralytion of S6 and Akt (threonine 308 and serine 473) in day 3 effector CD8+ T cells after a short (1h) *in vitro* stimulation with the OVA peptide (Fig 3B and 3C). Similar results were obtained when the phosphorylation of Akt and S6 was measured directly *ex vivo* (S2 Fig). As the activation of the Akt-mTOR axis was not affected in HES1-deficient day 3 effector CD8^+^ T cells, we further validated the lack of *Hes1* transcription in *Hes1*^Δ/Δ^ effector CD8^+^ T cells (S1A Fig). Our results suggest that the Notch signaling pathway influences the expression of other genes than *Hes1* and *Pten* to promote proper activation of the Akt signaling pathway and effector CD8^+^ T cell differentiation. Alternatively, Notch signaling may regulate Akt activation via a non-canonical pathway as shown in other experimental systems [23,24]. The identification of the direct Notch target genes in mature CD8^+^ T cells should help to understand how Notch signaling influences activation of the Akt-mTOR pathway during SLEC differentiation.

## Supporting information

S1 Fig Efficient deletion of *Hes1* in Hes1^Δ/Δ^ effector CD8^+^ T cells and decreased Akt phosphorylation in absence of Notch signalling. (A) *Hes1*^Δ/Δ^ effector CD8^+^ T cells do not transcribe *Hes1*. *Hes1*^fl/fl^ and Hes1^Δ/Δ^ OT-I T cells were adoptively transferred into congenic B6.SJL recipients (CD45.1^+^). One day later mice were infected with Lm-OVA. At day 3 post-infection with Lm-OVA, effector T cells (CD8^+^CD45.2^+^) were sorted to measure *Hes1* transcription using RT-qPCR. Naïve OT-I T cells were used as a positive control. (B) Efficient deletion of the *Hes1* gene in effector CD8^+^ T cells. *Hes1*^fl/fl^ and Hes1^Δ/Δ^ mice were infected with Lm-OVA, 7 days later OVA-specific effector CD8^+^ T cells (CD8^+^Tet-OVA^+^CD44^hi^), naïve endogenous CD8^+^ T cells (CD8^+^CD44^lo^) and CD4^+^ T cells were sorted for DNA extraction. Quantitative qPCR was performed to measure the extent of *Hes1* gene deletion using CD4^+^ T cells as a reference. (C) Reduction of Akt phosphorylation in absence of Notch signalling. N1N2^fl/fl^ and N1N2^Δ/Δ^ OT-I T cells (CD45.2^+^) were adoptively transferred into congenic B6.SJL recipients (CD45.1^+^). One day later mice were infected with Lm-OVA. At day 3 post-infection, splenocytes were rested in media for one hour before stimulation with the OVA peptide for one hour. Cells were fixed, permeabilized and stained to measure the phosphorylation of Akt in OVA-specific CD8^+^ T cells (CD8^+^CD45.2^+^). Endogenous (Endo; CD8^+^CD45.2^-^) cells were used as staining control. The bar graphs show the ratio of the MFI of OVA-specific CD8+ T cells over the endogenous CD8^+^ T cells. Statistical significance was determined using ANOVA (A) and Student’s t test (C).
(PDF)

S2 Fig HES1-deficient and sufficient effector CD8^+^ T cells show similar level of phosphorylation of S6 and Akt *ex vivo*. WT and HES1-deficient OT-I CD8^+^ T cells were adoptively transferred in B6.SJL mice followed by infection with Lm-OVA. At day 3 post-infection, cells were stained intracellularly to detect phospho-Akt (A) and phospho-S6 (B) in OVA-specific OT-I effectors (CD8^+^CD45.2^+^). Endogenous CD8^+^ T cells from the same recipient mice (CD8^+^CD45.2^-^) were used as a staining control. The bar graphs show the ratio of the MFI of OVA-specific CD8^+^ T cells over the endogenous CD8^+^ T cells. Two independent experiments with 5 mice per group. Statistical significance was determined using Student’s t test.
(PDF)

S1 File Statistical data. Individual data, mean, SD, SE and statistical tests for each experiment described in the manuscript.
(XLSX)
