# Supplementary material for: The Notch signaling pathway controls CD8+ T cell differentiation independently of the classical effector HES1
Source: PLoS One. 2019 Apr 5;14(4):e0215012. doi: 10.1371/journal.pone.0215012 (PMC6450647; doi:10.1371/journal.pone.0215012)
Supplement: S1 Fig — (A) Hes1Δ/Δ effector CD8+ T cells do not transcribe Hes1. Hes1fl/fl and Hes1Δ/Δ OT-I T cells were adoptively transferred into congenic B6.SJL recipients (CD45.1+). One day later mice were infected with Lm-OVA. At day 3 post-infection with Lm-OVA, effector T cells (CD8+CD45.2+) were sorted to measure Hes1 transcription using RT-qPCR. Naïve OT-I T cells were used as a positive control. (B) Efficient deletion of the Hes1 gene in effector CD8+ T cells. Hes1fl/fl and Hes1Δ/Δ mice were infected with Lm-OVA, 7 days later OVA-specific effector CD8+ T cells (CD8+Tet-OVA+CD44hi), naïve endogenous CD8+ T cells (CD8+CD44lo) and CD4+ T cells were sorted for DNA extraction. Quantitative qPCR was performed to measure the extent of Hes1 gene deletion using CD4+ T cells as a reference. (C) Reduction of Akt phosphorylation in absence of Notch signalling. N1N2fl/fl and N1N2Δ/Δ OT-I T cells (CD45.2+) were adoptively transferred into congenic B6.SJL recipients (CD45.1+). One day later mice were infected with Lm-OVA. At day 3 post-infection, splenocytes were rested in media for one hour before stimulation with the OVA peptide for one hour. Cells were fixed, permeabilized and stained to measure the phosphorylation of Akt in OVA-specific CD8+ T cells (CD8+CD45.2+). Endogenous (Endo; CD8+CD45.2-) cells were used as staining control. The bar graphs show the ratio of the MFI of OVA-specific CD8+ T cells over the endogenous CD8+ T cells. Statistical significance was determined using ANOVA (A) and Student’s t test (C). (PDF) [file pone.0215012.s001.pdf]

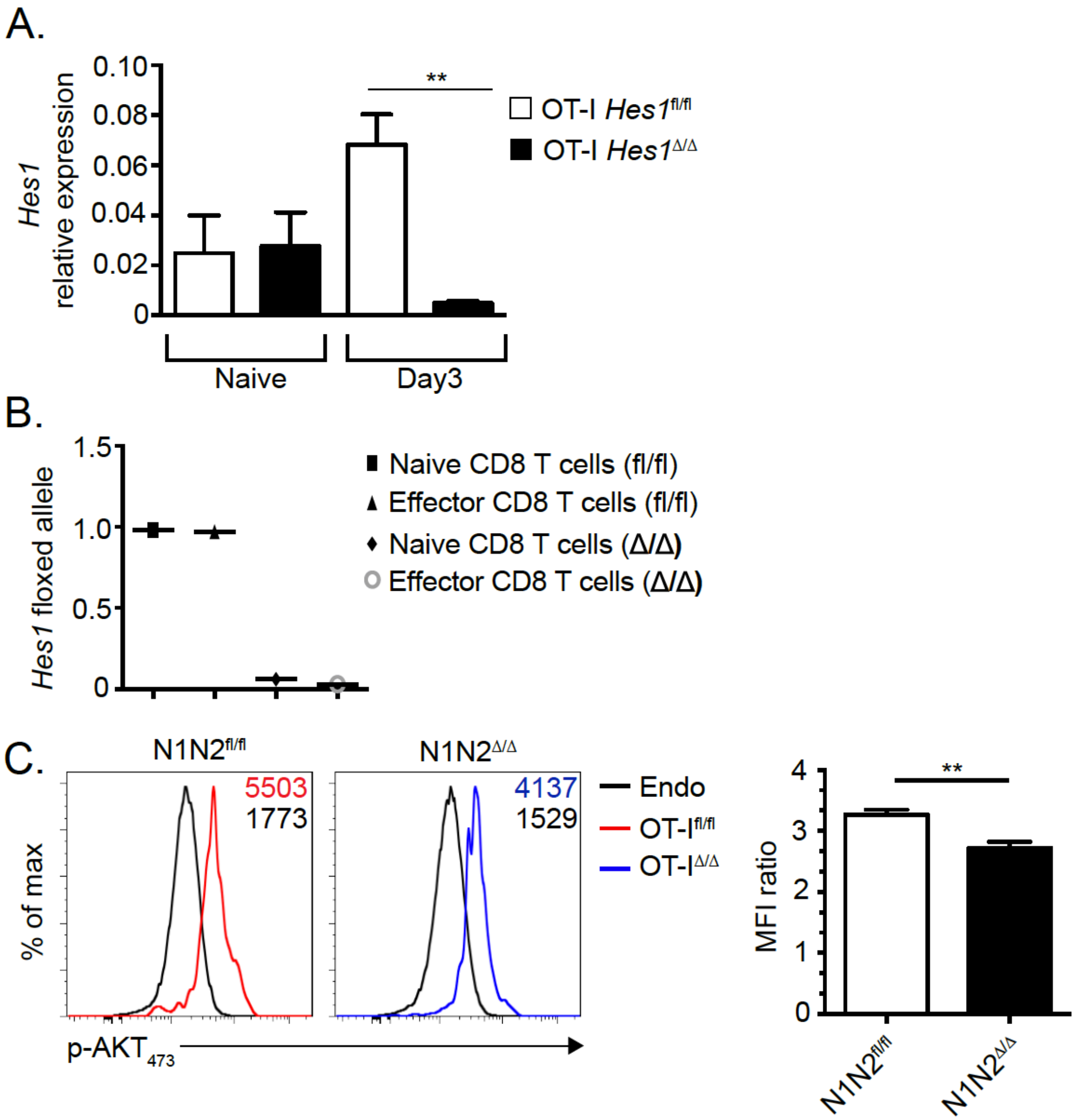

Supplemental Figure 1. (**A**) *Hes1*$^{\Delta/\Delta}$ effector CD8^{+} T cells do not transcribe *Hes1*. *Hes1*$^{fl/fl}$ and Hes1$^{\Delta/\Delta}$ OT-I T cells were adoptively transferred into congenic B6.SJL recipients (CD45.1^{+}). One day later mice were infected with Lm-OVA. At day 3 post-infection with Lm-OVA, effector T cells (CD8^{+}CD45.2^{+}) were sorted to measure *Hes1* transcription using RT-qPCR. Naïve OT-I T cells were used as a positive control. (**B**) Efficient deletion of the *Hes1* gene in effector CD8^{+} T cells. *Hes1*$^{fl/fl}$ and Hes1$^{\Delta/\Delta}$ mice were infected with Lm-OVA, 7 days later OVA-specific effector CD8^{+} T cells (CD8^{+}Tet-OVA^{+}CD44^{hi}), naïve endogenous CD8^{+} T cells (CD8^{+}CD44^{lo}) and CD4^{+} T cells were sorted for DNA extraction. Quantitative qPCR was performed to measure the extent of *Hes1* gene deletion using CD4^{+} T cells as a reference. (**C**) Reduction of Akt phosphorylation in absence of Notch signalling. N1N2$^{fl/fl}$ and N1N2$^{\Delta/\Delta}$ OT-I T cells (CD45.2^{+}) were adoptively transferred into congenic B6.SJL recipients (CD45.1^{+}). One day later mice were infected with Lm-OVA. At day 3 post-infection, splenocytes were rested in media for one hour before stimulation with the OVA peptide for one hour. Cells were fixed, permeabilized and stained to measure the phosphorylation of Akt in OVA-specific CD8^{+} T cells (CD8^{+}CD45.2^{+}). Endogenous (Endo; CD8^{+}CD45.2^{-}) cells were used as staining control. The bar graphs show the ratio of the MFI of OVA-specific CD8+ T cells over the endogenous CD8^{+} T cells. Statistical significance was determined using ANOVA (A) and Student's t test (C).
