# Supplementary material for: The Notch signaling pathway controls CD8+ T cell differentiation independently of the classical effector HES1
Source: PLoS One. 2019 Apr 5;14(4):e0215012. doi: 10.1371/journal.pone.0215012 (PMC6450647; doi:10.1371/journal.pone.0215012)
Supplement: S2 Fig — WT and HES1-deficient OT-I CD8+ T cells were adoptively transferred in B6.SJL mice followed by infection with Lm-OVA. At day 3 post-infection, cells were stained intracellularly to detect phospho-Akt (A) and phospho-S6 (B) in OVA-specific OT-I effectors (CD8+CD45.2+). Endogenous CD8+ T cells from the same recipient mice (CD8+CD45.2-) were used as a staining control. The bar graphs show the ratio of the MFI of OVA-specific CD8+ T cells over the endogenous CD8+ T cells. Two independent experiments with 5 mice per group. Statistical significance was determined using Student’s t test. (PDF) [file pone.0215012.s002.pdf]

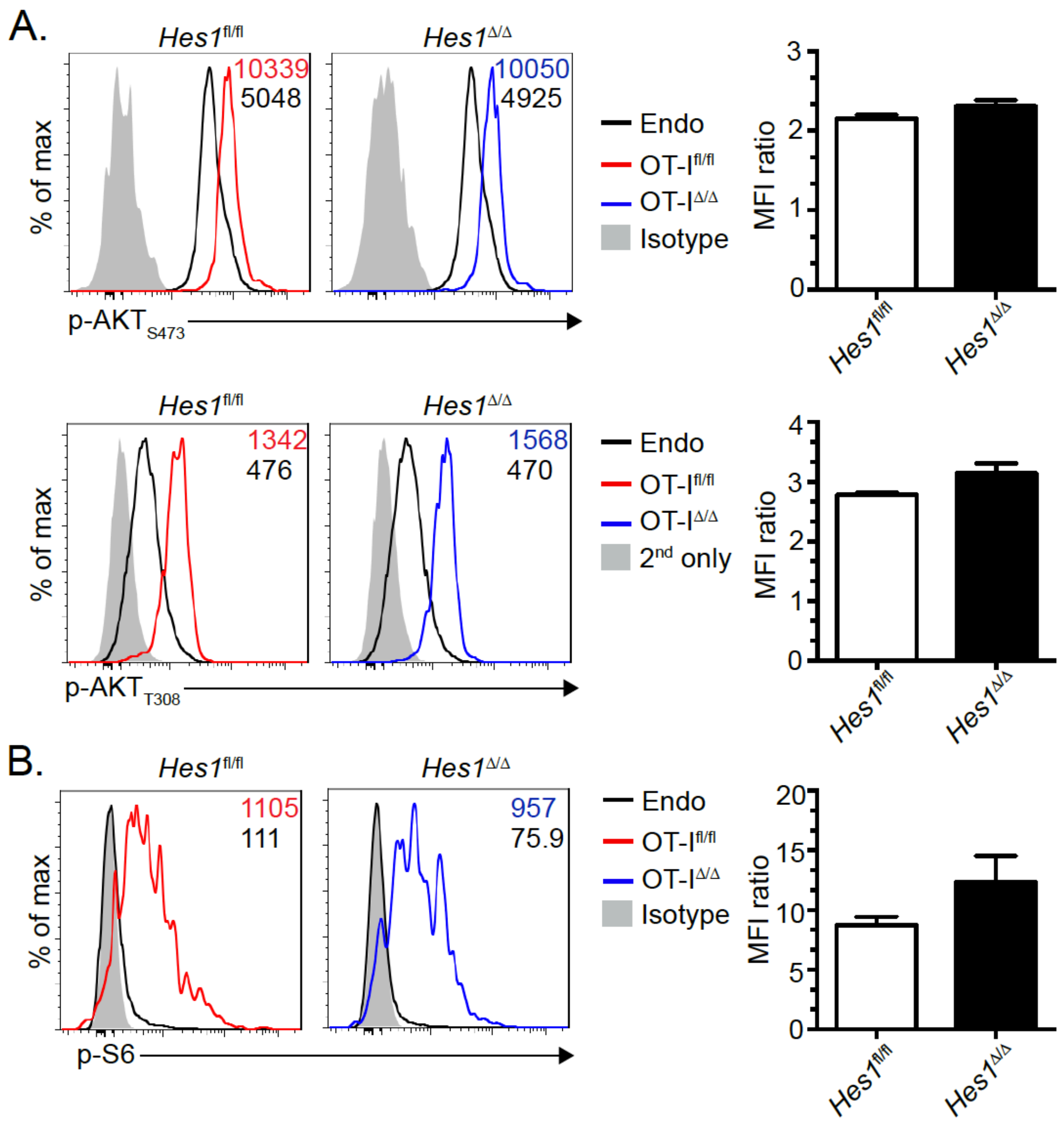

Supplemental Figure 2. HES1-deficient and sufficient effector $CD8^+$ T cells show similar level of phosphorylation of S6 and Akt *ex vivo*. WT and HES1-deficient OT-I $CD8^+$ T cells were adoptively transferred in B6.SJL mice followed by infection with Lm-OVA. At day 3 post-infection, cells were stained intracellularly to detect phospho-Akt (A) and phospho-S6 (B) in OVA-specific OT-I effectors ($CD8^+CD45.2^+$). Endogenous $CD8^+$ T cells from the same recipient mice ($CD8^+CD45.2^-$) were used as a staining control. The bar graphs show the ratio of the MFI of OVA-specific $CD8^+$ T cells over the endogenous $CD8^+$ T cells. Two independent experiments with 5 mice per group. Statistical significance was determined using Student's t test.
